# Calcium-Dependent Cytosolic Phospholipase A2α as Key Factor in Calcification of Subdermally Implanted Aortic Valve Leaflets

**DOI:** 10.3390/ijms23041988

**Published:** 2022-02-11

**Authors:** Antonella Bonetti, Magali Contin, Federica Tonon, Maurizio Marchini, Fulvia Ortolani

**Affiliations:** Department of Medicine, University of Udine, I-33100 Udine, Italy; antonella.bonetti@uniud.it (A.B.); magali.contin@uniud.it (M.C.); ftonon@units.it (F.T.); maurizio.marchini@uniud.it (M.M.)

**Keywords:** cPLA2α, valve calcification, aortic valve leaflets, aortic valve interstitial cells, subdermal model, ultrastructure, immunogold labelling

## Abstract

Calcium-dependent cytosolic phospholipase A2α (cPLA2α) had been previously found to be overexpressed by aortic valve interstitial cells (AVICs) subjected to in vitro calcific induction. Here, cPLA2α expression was immunohistochemically assayed in porcine aortic valve leaflets (iAVLs) that had undergone accelerated calcification subsequent to 2- to 28-day-long implantation in rat subcutis. A time-dependent increase in cPLA2α-positive AVICs paralleled mineralization progression depending on dramatic cell membrane degeneration with the release of hydroxyapatite-nucleating acidic lipid material, as revealed by immunogold particles decorating organelle membranes in 2d-iAVLs, as well as membrane-derived lipid byproducts in 7d- to 28d-iAVLs. Additional positivity was detected for (i) pro-inflammatory IL-6, mostly exhibited by rat peri-implant cells surrounding 14d- and 28d-iAVLs; (ii) calcium-binding osteopontin, with time-dependent increase and no ossification occurrence; (iii) anti-calcific fetuin-A, mostly restricted to blood plasma within vessels irrorating the connective envelopes of 28d-iAVLs; (iv) early apoptosis marker annexin-V, limited to sporadic AVICs in all iAVLs. No positivity was found for either apoptosis executioner cleaved caspase-3 or autophagy marker MAP1. In conclusion, cPLA2α appears to be a factor characterizing AVL calcification concurrently with a distinct still uncoded cell death form also in an animal model, as well as a putative target for the prevention and treatment of calcific valve diseases.

## 1. Introduction

Calcific aortic valve stenosis (CAVS) is regarded as a multifactorial disorder, whose pathogenesis includes mechanical stress [1], extracellular matrix remodelling [2], lipid accumulation and/or alteration [3], oxidative stress [4], inflammation [5], a decrease in anti-calcific factors [6], mineral imbalance [7], angiogenesis [8], and heterotopic ossification, depending on osteoblastic transdifferentiation of aortic valve interstitial cells (AVICs) [9]. Except for the unquestionable concept that ossification implies calcification but not vice versa, as matter of fact the most common event preceding and likely triggering all calcific aortic valve diseases is AVIC alteration and/or death in its various forms as reported [10,11,12,13]. In addition to (i) calcium-binding osteopontin (OPN), osteocalcin, and osteonectin [14,15], (ii) proteoglycans rich in acidic glycosaminoglycan lateral chains [16], and (iii) alkaline phosphatase (ALP) [17], increasing attention has been focused on the role of phospholipases (PLAs) in CAVS onset, with a particular focus on circulating lipoprotein-associated PLA isoforms [18,19]. Conversely, there is still little research on the involvement of calcium-dependent cytosolic PLA2α (cPLA2α) in biomineralization processes, including calcific valve diseases [20,21,22,23]. cPLA2α, also known as group IVA PLA2 or PLA2G4A, is a constitutively expressed enzyme that catalyzes the hydrolysis of membrane phospholipids to arachidonic acid and lysophospholipids, with subsequent downstream production of bioactive lipids including eicosanoids. Therefore, cPLA2α may be reasonably suspected to be a triggering factor in the peculiar lipid-release-associated degeneration that was found to affect AVICs subjected to calcification in both an in vivo environment, after implantation of porcine aortic valve leaflets (AVLs) in the rat subdermis [24,25,26,27,28,29] and in in vitro conditions [28,29,30,31,32], after cell stimulation with pro-calcific agents. In short, both these experimental conditions gave rise to a sequence of analogous pro-calcific degenerative stages including (i) widespread cell membrane dissolution, with concurrent organelle disappearance and formation of intracytoplasmic dense bodies, (ii) their merging into an acidic phthalocyanine-positive material (PPM), (iii) PPM outward shift forming peripheral phthalocyanine-positive layers (PPLs), clearly recognizable as major hydroxyapatite (HA) crystal nucleators, and (iv) release of PPL-lined vesicular bodies in their turn budding real *calcospherulae*. Of note, HA-nucleating anionic phospholipids were also identified in CAVS-affected human AVLs analyzed using Raman microspectroscopy [33]. Concerning the in vitro pro-calcific models as above, cPLA2α expression by cultured AVICs was found to occur, with time-dependent enzyme increase paralleling calcification advancement [32]. To corroborate the hypothesis of cPLA2α involvement also in valve tissue calcification, in the present study, enzyme expression was immunohistochemically assayed in porcine AVLs subdermally implanted in rats for 2 to 28 days (2d-iAVLs, 7d-iAVLs, 14d-iAVLs, and 28d-iAVLs) versus native, non-implanted ones (nAVLs). In iAVLs, increasing numbers of AVICs resulted to express cPLA2α in a time-dependent manner, closely paralleling the progression of the aforementioned AVIC pro-calcific degeneration.

## 2. Results

Positivity for markers immunohistochemically assayed in nAVLs and iAVLs is reported in Table 1.

### 2.1. cPLA2α Expression and iAVL Calcification

**Figure 1:**
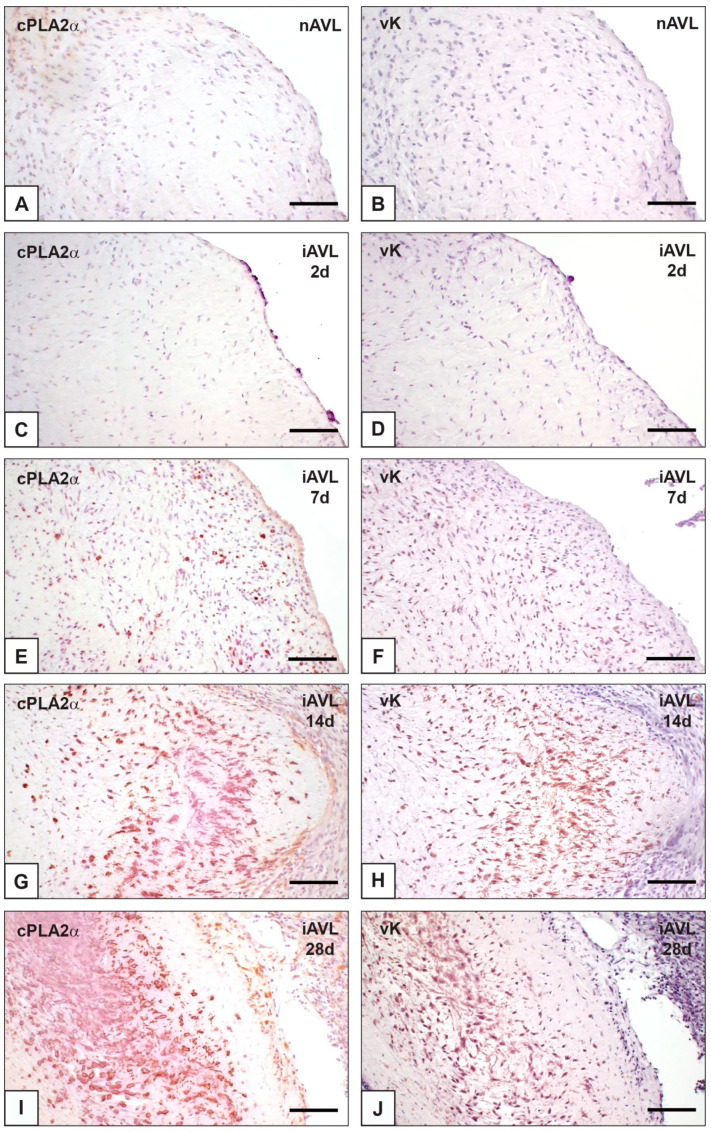
Histological sections of porcine aortic valve leaflets in the native state (nAVL) or subdermally implanted into rat subcutis for 2 to 28 days (iAVL 2d, iAVL 7d, iAVL 14d, and iAVL 28d) after the immunohistochemical detection of cytosolic phospholipase A2α (cPLA2α; left column) matched against para-serial sections subjected to von Kossa silver reaction (vK) for calcium binding site identification (right column). (**A**–**D**) Negligible positivity for cPLA2α showed by both nAVL (**A**) and iAVL 2d (**C**) vs. negativity for vK showed by both nAVL (**B**) and iAVL 2d (**D**). (**E**–**J**) Time-dependent increase in cPLA2α-positive valvular cells in iAVL 7d (**E**), iAVL 14d (**G**), and iAVL 28d (**I**) vs. parallel increase in vK-positive ones (**F**,**H**,**J**). Bar: 0.5 mm.

**Figure 2:**
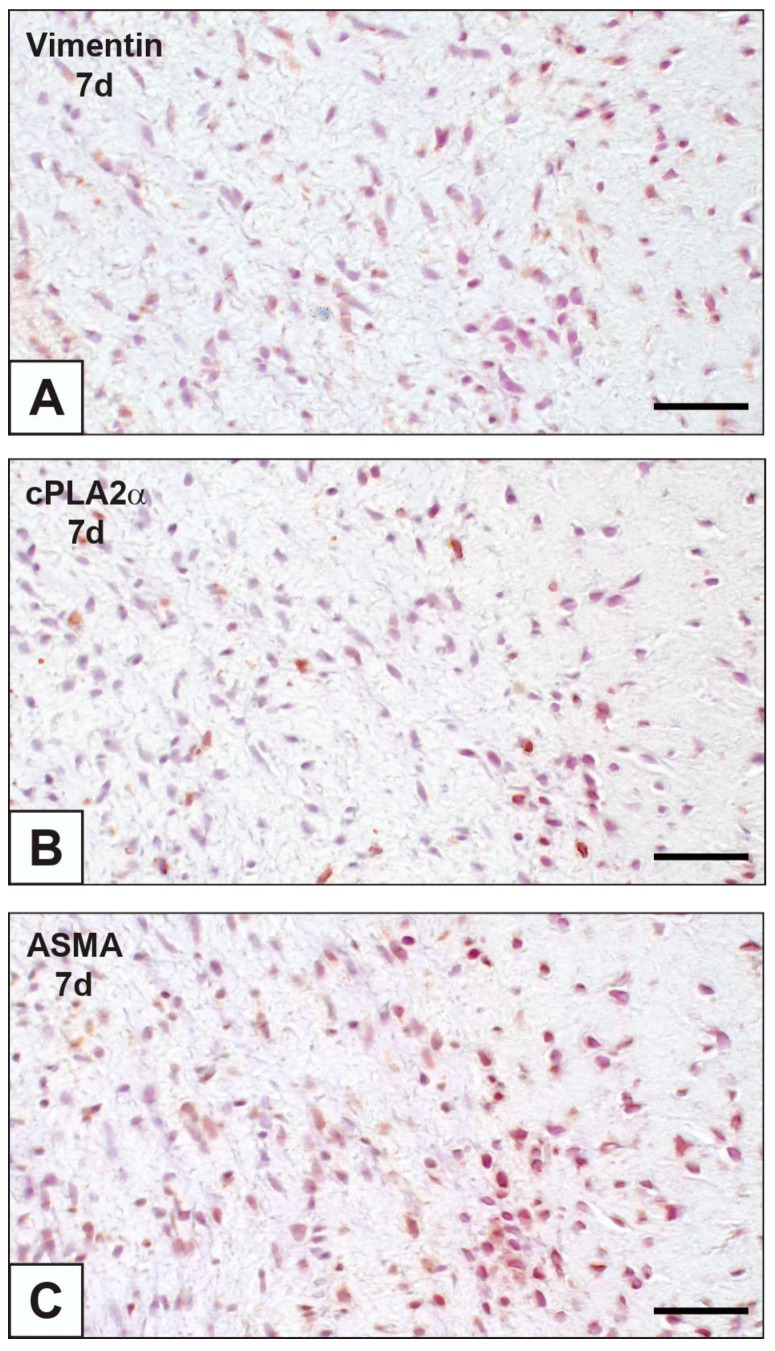
Comparison between para-serial histological sections of an aortic valve leaflet implanted into rat subcutis for 7 days (7d) after immunohistochemical detection of (**A**) vimentin, (**B**) cytosolic phospholipase A2α (cPLA2α), and (**C**) α-smooth muscle actin (ASMA). Co-localization in superimposable areas of cPLA2α-positive valvular cells (**B**) with both vimentin-positive (**A**) and ASMA-positive (**C**) ones. Bar: 0.25 mm.

**Figure 3:**
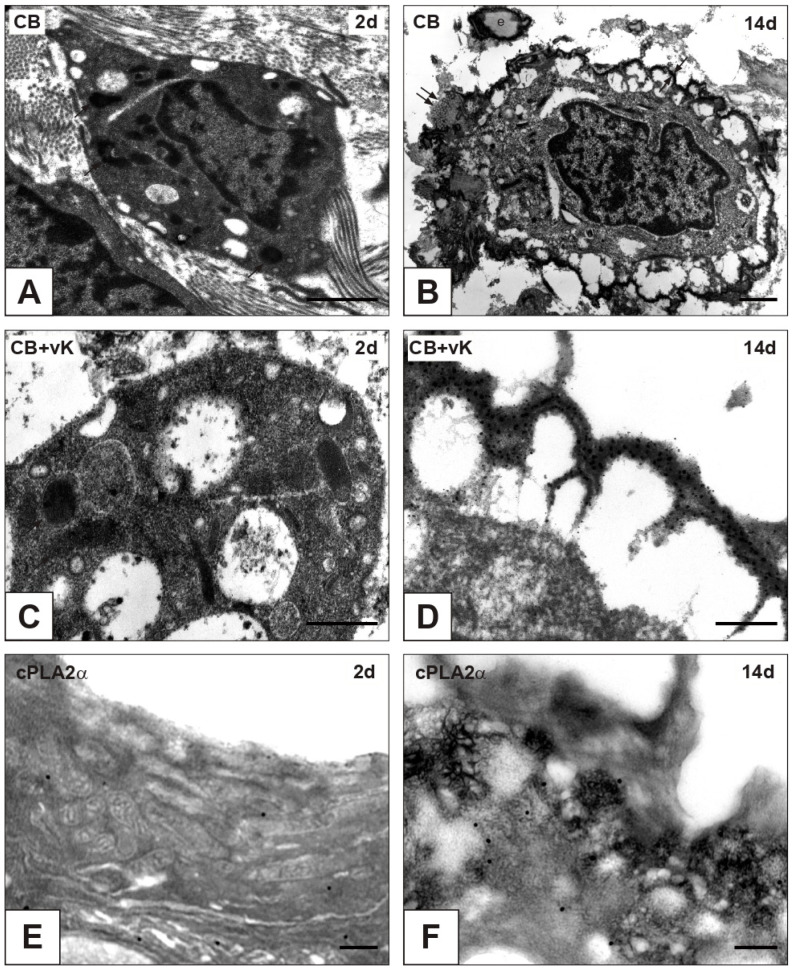
Thin sections of aortic valve leaflets implanted into rat subcutis for 2 days (2d; left column) vs. those implanted for 14 days (14d; right column). (**A**,**B**) Pre-embedding reaction with phthalocyanine cuprolinic blue (CB) showing CB-reactive dense bodies (arrows) in a valvular cell at early degeneration stage vs. appearance of a CB-positive, membrane-derived pericellular layer (counterposed arrows) embedding outside collagen fibrils (double arrows) and elastin fibers (e) at late degeneration stage. (**C**,**D**) CB-positive dense bodies (arrows in **C**) showing negligible positivity to additional post-embedding von Kossa reaction (vK) vs. a CB-positive pericellular layer exhibiting large silver particle superimposition after vK (**D**). (**E**,**F**) Immunogold labelling of cytosolic phospholipase A2α (cPLA2α) showing selective decoration of mitochondrial membranes in a valvular cell at early degeneration stage (**E**) vs. large decoration of a pericellular layer at late degeneration stage (**F**). Bar: 1 μm (**A**,**B**); 0.5 μm (**C**,**E**,**F**); 0.25 μm (**D**).

On paraffin sections of nAVLs, immunopositivity to cPLA2α was restricted to sporadic AVICs (Figure 1A), whilst no positivity to von Kossa silver staining (vK) resulted in adjacent sections (Figure 1B). Likewise, only occasional immunoreactivity to cPLA2α and negativity to vK were found for 2d-iAVLs (Figure 1C,D). Conversely, marked increase in cPLA2α-positive AVICs resulted for 7d-iAVLs at the *tunica fibrosa* and *tunica spongiosa* levels (Figure 1E), with a few cPLA2α-positive endothelial cells also being appreciable. On adjacent sections, increased positivity to vK was also apparent, especially at the AVIC edges (Figure 1F). An additional increase in cPLA2α-positive AVICs and endothelial cells resulted for 14d-iAVLs (Figure 1G), with a parallel increase in vK-positive AVICs being appreciable on adjacent sections (Figure 1H). A further increase in both cPLA2α-positive (Figure 1I) and vK-reactive (Figure 1J) valvular cells resulted for 28d-iAVLs. In the latter, positivity to vK was also shown by a lot of heterogeneously sized microprecipitates consisting of vesicular byproducts derived from AVIC pro-calcific degeneration. cPLA2α-positive AVICs showed positivity for vimentin and α-smooth muscle actin (ASMA) too (Figure 2A–C), suggesting all AVIC types, i.e., fibroblasts, myofibroblasts, and smooth muscle cells, to be directly involved in enzyme expression. As expected, immunopositivity to vimentin was also shown by the endothelial cells, some of which showed additional positivity for ASMA. Several endothelial cells were also positive for the macrophage marker CD68, whereas no reactivity resulted for cells populating the leaflet *interstitium* (not shown). On thin sections, AVICs from 2d-iAVLs showed organelle degeneration, with the appearance of intracellular phthalocyanine-positive dense bodies readily recognizable as early degenerative features (Figure 3A,C). Immunogold labelling confirmed cPLA2α to be mildly expressed in some valvular cells, with gold particles mostly decorating still recognizable mitochondrial membranes (Figure 3E). Occasionally, gold particles were observed on nuclear envelopes, whereas plasma membranes were not decorated. As previously described, in 7d- to 28d-iAVLs most mineralizing AVICs were lined by blebbing PPLs, with PPL-derived material expanding outwards embedding the nearby extracellular matrix components (Figure 3B). PPLs were selective sites for metallic silver particle precipitation after post-embedding vK reactions (Figure 3D), consistent with their role as HA nucleators. Such pro-calcific lipid material was electively decorated by gold particles after immunogold labelling reactions against cPLA2α (Figure 3F).

### 2.2. Interleukin-6 (IL-6) Expression

**Figure 4:**
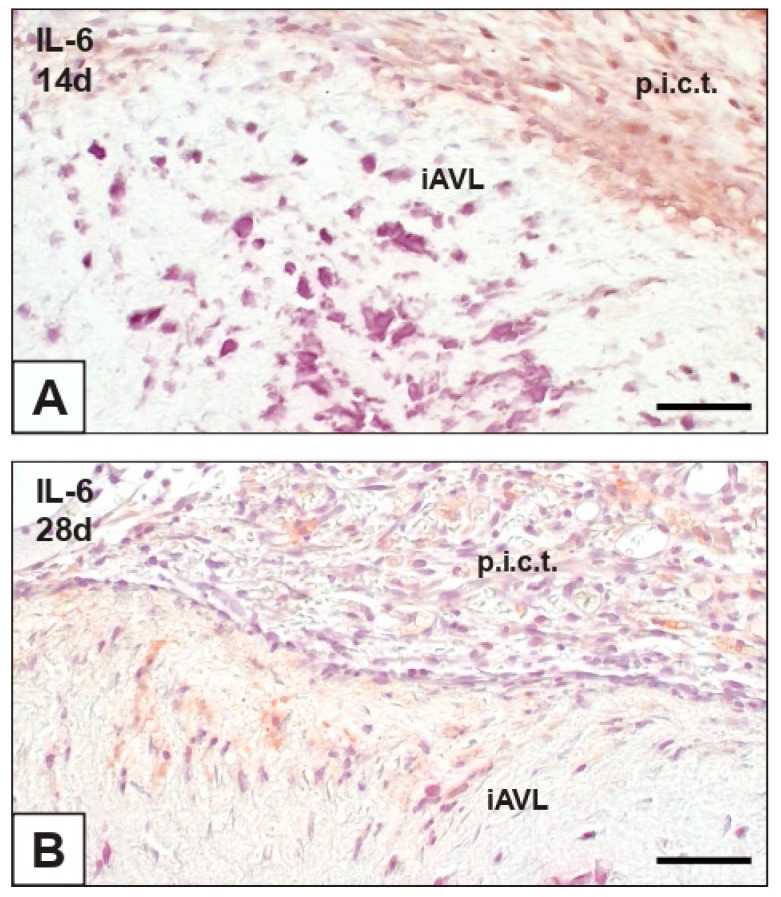
Histological section of an aortic valve leaflet implanted into rat subcutis for 14 days (iAVL 14d) vs. another implanted for 28 days (iAVL 28d) after immunohistochemical detection of interleukin-6 (IL-6). (**A**) Positivity of valvular endothelial cells and host’s cells of the peri-implant connective tissue (p.i.c.t.). (**B**) Positivity of valvular subendothelial cells and host’s cells of the peri-implant connective tissue (p.i.c.t.). Bar: 0.25 mm.

The immunohistochemical assay for IL-6 revealed that this pro-inflammatory cytokine was not expressed in nAVLs and 2d-iAVLs, except for some round mononuclear and polymorphonuclear cells adhering to the surface of the latter (not shown). Conversely, immunopositivity to IL-6 slightly increased in 7d- to 28d-iAVLs, involving endothelial (Figure 4A) and sub-endothelial cells (Figure 4B) mostly at the *tunica fibrosa* level. Greater numbers of IL-6-positive cells were observed in the rat connective tissue enveloping both 14d- and 28d-iAVLs (Figure 4A,B).

### 2.3. Expression of OPN and Fetuin-A

**Figure 5:**
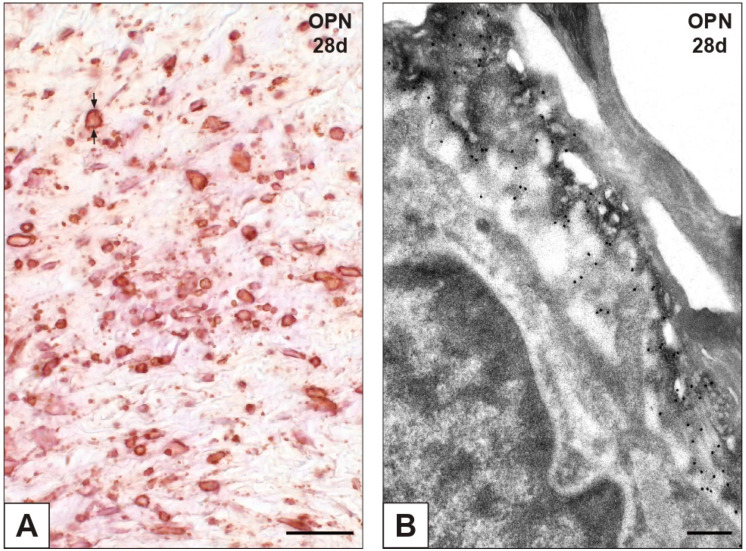
Immunohistochemical detection of osteopontin (OPN) in aortic valve leaflets implanted into rat subcutis for 28 days (28d). (**A**) Histological section showing positive valvular cells exhibiting marked reactivity at their edges (counterposed arrows). (**B**) Thin section subjected to immunogold labelling showing a membrane-derived pericellular layer decorated by gold particles. Bar: 0.25 mm (**A**); 0.25 μm (B).

**Figure 6:**
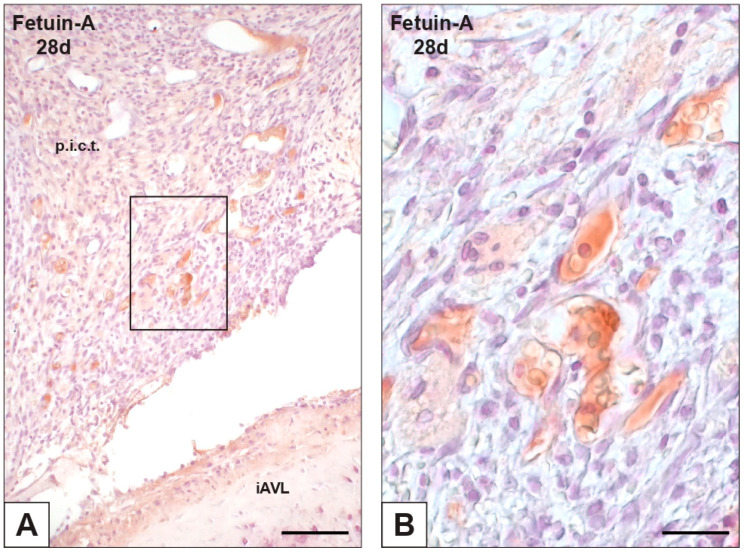
Histological section of an aortic valve leaflet implanted into the rat subcutis for 28 days (iAVL 28d) after immunohistochemical detection of fetuin-A. (**A**) Positivity of blood plasma of vessels irrorating the peri-implant connective tissue (p.i.c.t.), with no positivity showed by valvular cells. (**B**) Magnification of the immunopositive vessels in the framed area in (**A**). Bar: 0.5 mm (**A**); 0.1 mm (**B**).

Immunopositivity to calcium-binding OPN was observed for some AVICs and endothelial cells in both nAVLs and 2d-iAVLs (not shown), whereas remarkably increased numbers of reactive cells were apparent in 7d- to 28d-iAVLs. After days 14 and 28 of implantation, immunopositivity to OPN was more marked at the AVIC edges (Figure 5A), consistently with remarkable decoration of peripheral PPLs by gold particles after immunogold labelling reactions (Figure 5B). Immunopositivity to the anti-calcific protein fetuin-A was only observed for sporadic AVICs in the nAVLs, whereas no positivity resulted for any of the iAVLs (not shown). Of interest, positivity to fetuin-A was exhibited by the blood plasma of vessels irrorating the rat connective tissue enveloping 14d- and, to a greater extent, 28d-iAVLs (Figure 6A,B).

### 2.4. Expression of Cell Death Markers

**Figure 7:**
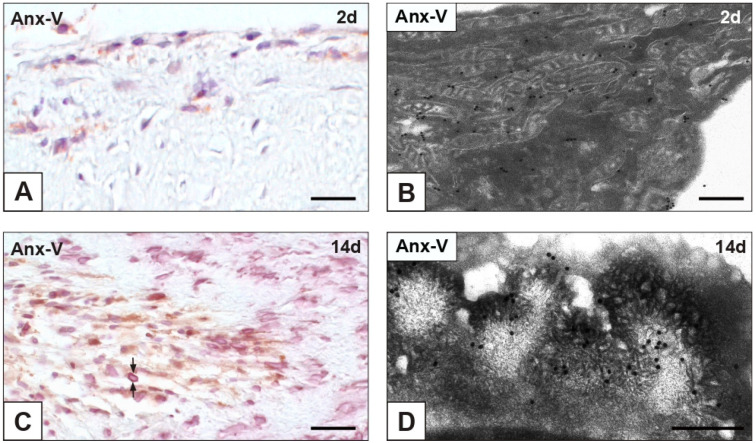
Immunohistochemical detection of annexin-V (Anx-V) in aortic valve leaflets implanted into rat subcutis for 2 days (2d; line above) vs. 14 days (14d; line below). (**A**) Histological section showing immunopositivity for sporadic valvular cells. (**B**) Thin section subjected to immunogold labelling showing mitochondrial membranes decorated by gold particles in a valvular cell at early degeneration stage. (**C**) Histological section showing increased numbers of immunopositive valvular cells, with marked reactivity at their edges (counterposed arrows). (**D**) Thin section subjected to immunogold labelling showing a membrane-derived pericellular layer decorated by gold particles in a valvular cell at late degeneration stage. Bar: 0.25 mm (**A**,**C**); 0.25 μm (**B**,**D**).

Negligible or no immunopositivity to early apoptosis marker annexin-V (Anx-V) resulted for nAVLs and 2d-iAVLs (Figure 7A). In the latter, immunogold particles were found to decorate cell membranes, and, to a greater extent, mitochondrial membranes (Figure 7B). Starting from 7 implantation days, a slight increase in Anx-V-positive cells was observed, remaining unchanged up to 28 implantation days. Reactivity was particularly marked at cell edges in 14d- (Figure 7C) and 28d-iAVLs, consistently with labelling by gold particles of PPLs lining mineralized AVICs (Figure 7D). No immunopositivity to either apoptosis-executioner cleaved caspase-3 or autophagy marker MAP1 was found for all the examined nAVLs and iAVLs (not shown).

## 3. Discussion

The pathogenesis of calcific heart valve diseases including CAVS requires further elucidation, as it still remains unclear (i) which pro-calcific factors are directly involved, (ii) whether they play a role in promoting a pro-calcific AVIC death, and (iii) the real form of such cell death. In order to add information on these issues, the present study was conducted on AVLs subjected to experimental calcification after xenogeneic subdermal implantation, since this animal model was found to provide a faithful simulation of actual pathological conditions [34].

### 3.1. cPLA2α as Key Pro-Calcific Trigger

In the experimental pro-calcific model used, the immunohistochemical assays revealed cPLA2α expression by mineralizing AVICs to be strictly concurrent with iAVL calcification stages. The observed time-dependent increase in enzyme expression paralleled the onset and advancement of iAVL calcification, which occurred according to the multi-step degenerative pattern previously detected for the same experimental pro-calcific conditions in vivo, i.e., the xeno-implantation animal model [24,25,26,27,28,29], as well as in vitro pro-calcific conditions, i.e., stimulation of cultured AVICs with elevated inorganic phosphate levels and other enhancing agents [28,29,30,31,32]. The fact that cPLA2α resulted to be expressed by AVICs in the context of these two radically different experimental pro-calcific models provides compelling evidence that such enzyme expression is to be ascribed to a specific cell-death-associated mineralization process rather than a casual effect, depending on a given artificial manipulation. This concept is furtherly supported by the fact that analogous ultrastructural features were found to affect AVICs in pathologically mineralizing human AVLs [28]. Hence, the future assessment of cPLA2α expression in CAVS seems mandatory. The ultrastructural identification in early mineralizing AVICs of anti-cPLA2α gold particles mainly at the level of mitochondrial membranes suggests that the known enzyme translocation to plasmamembranes and nuclear membranes had not yet occurred. At more advanced stages, anti-cPLA2α gold particles appeared to decorate intracytoplasmic PPM and peripheral PPLs, confirming such a lipid material to originate from enzymatic membrane degradation and suggesting that the enzyme becomes entrapped therein. Since cPLA2α synthesis is known to be elicited by pro-inflammatory mediators [35,36], the increased enzyme expression observed for iAVLs may depend on the establishment of a pro-inflammatory environment due to the host’s cell-mediated and/or humoral responses to the xeno-implant [37], considering that its immunogenicity is known to be not entirely neutralized by the mild glutaraldehyde fixation used in the subdermal model [38,39]. This assumption is consistent with the observed adhesion of rat defense cells to the iAVL surface and positivity to pro-inflammatory IL-6 for a lot of other host’s cells populating the peri-implant connective tissue. In addition, a few endothelial cells and subendothelial AVICs also showed reactivity to IL-6 in mineralizing 14d- and 28d-iAVLs, suggesting a responsivity of the implant towards the surrounding environment. In contrast with previous investigations [40], no entering of host-derived, CD68-positive monocytes/macrophages was detected, likely due to the glutaraldehyde-dependent crosslinking of tissue components preventing cell migration across the extracellular matrix. Consistently, round mononuclear and polymorphonuclear cells were just found to adhere to the iAVL surfaces, often being entrapped inside a fibrin-like material. Of note, some CD68-positive endothelial cells covering mineralized iAVLs were found to co-localize with those positive for cPLA2α, suggesting a direct involvement of the enzyme in endothelium activation [41]. Since some endothelial cells also showed immunopositivity to ASMA, it might be argued that an ongoing inflammation-dependent endothelial-to-mesenchymal transition took place. It remains unclear whether this transdifferentiation process contributes to iAVL calcification or may represent an attempt to counteract it. In addition to inflammation, glutaraldehyde-induced hypoxia might be an alternative or contributory cause of enhanced cPLA2α expression, consistently with the reported enzyme increase in mice subjected to induced cerebral ischemia [42]. Besides increased cPLA2α expression, concurrent enzyme activation was consistent with (i) occurrence of massive cell membrane lysis resulting in the described PPM/PPL formation and (ii) no cPLA2α inactivation by glutaraldehyde fixation, as previously found for calcification-related ALP [43], consistently with possible cPLA2α-mediated ALP downstream activation [44]. As in pathologically calcified heart valves [14,45,46,47,48], calcification-related OPN expression was immunohistochemically detected for the examined mineralizing iAVLs, with more marked positivity resulting at PPL level, as clearly confirmed by the immunogold labelling reactions. Although OPN is known to inhibit HA crystal growth or somehow control crystal size and shape [48], protein dephosphorylation might have occurred because of preserved ALP activity [49,50]. Consequent loss of OPN anti-calcific effect mainly at the level of pericellular PPLs is consistent with their role as major HA nucleators. Despite the fact that the increase in OPN expression was reported to correlate with bone formation during valve calcification, also including the co-presence of bone marrow or cartilage [51,52,53], it should be pointed out that in this study, as well as our previous investigations on AVIC calcification, osseous or cartilaginous ectopic foci were never encountered [24,25,26,27,28,29,30,31,32,33]. Actually, heterotopic bone formation was histologically shown in a very low percentage of explanted CAVS-affected valves [54,55], strongly suggesting that ossification is not a *sine qua non* process underlying valve calcification, but rather a more or less frequent epiphenomenon. Although anti-calcific fetuin-A was reported to be more expressed in CAVS-affected AVLs compared with healthy ones [56], in this study, no AVICs from iAVLs showed positivity to such protein. Whilst fetuin-A serum levels have not been univocally related to the risk of valve calcification [7,57], this circulating protein was reported to act as a protective agent in systemic inflammation [58]. Since marked positivity resulted for blood plasma of vessels irrorating the connective tissue enveloping 28d-iAVLs, such an increase in circulating fetuin-A might be related to inflammation lowering as a late host’s response to the xeno-implant.

### 3.2. Non-Canonical Cell Death in iAVL Calcification

The typical pro-calcific degenerative steps previously reported to affect AVICs in both in vivo and in vitro experimental conditions [24,25,26,27,28,29,30,31,32], as well as in the present study, constitute a complex morphological pattern that can be viewed as a hallmark of a novel form of lipid-release-associated cell death not fitting into one of the forms of programmed or non-programmed cell death as coded so far [59]. Being that Anx-V was identified as an early apoptosis marker [60], the positivity observed for some AVICs in iAVLs means it cannot be excluded that an apoptotic process is associated with valve calcification. However, negativity to apoptosis executioner cleaved caspase-3 strongly supports the concept that, though triggered, apoptosis is not completed in the iAVLs, as previously discussed [27]. This assumption is further supported by the resulting cPLA2α increase, given that caspase-3 was reported to cause cPLA2α inhibition [61]. It is of note that no apoptosis execution resulted for degenerating AVICs cultured under pro-calcific conditions [31]. Bearing in mind that both Anx-V and cPLA2α have affinity for anionic phospholipids [62], either antagonism or synergy between these two proteins might take place in the context of a substrate depletion mechanism [62,63], in some way influencing calcium uptake. Indeed, Anx-V was reported to act as a specific calcium channel promoting calcium influx into mineralizing chondrocytes and chondrocyte-derived matrix vesicles [64,65]. Although autophagy promotes cell homeostasis and survival through selective degradation/recycling of cytoplasm components, cell demise can result from excessive autophagic processes, as reported for actual CAVS [11]. However, negativity to autophagy marker MAP1 for all the examined AVLs suggests that not even autophagic cell death is involved in iAVL calcification. It is worth noting that recent evidence of cPLA2α-mediated inhibition of autophagy in experimental conditions inducing neuronal death is consistent with the present results [66]. In addition, cultured AVICs revealed no orthodox autophagy occurrence under pro-calcific conditions and the activation of atypical, non-lysosomal autophagic processes under sub-mineralizing conditions [31]. Although in the absence of specific markers for unambiguous detection of cell oncosis (necrosis), the ultrastructural alterations associated with this form of cell death, such as cell swelling and/or release of cellular content into the surrounding extracellular matrix, were never found in the examined iAVLs, leading its occurrence to be excluded too.

In conclusion, cPLA2α was also shown to be involved in AVIC calcification in an animal model, showing a close relationship with a peculiar pro-calcific cell death, presumably including its action as a specific trigger, in a context deserving further investigation to shed more light on aortic valve calcific diseases and possibly to propose the enzyme as a target for their prevention and treatment.

## 4. Materials and Methods

### 4.1. Sampling

Histological sections were obtained from paraformaldehyde-fixed, paraffin-embedded (i) native AVLs from pig hearts retrieved at slaughtering (nAVLs; *n* = 3) and (ii) AVLs as in (i) subjected to accelerated calcific induction by subdermal implantation in rats according to Schoen and colleagues [67] for 2, 7, 14, and 28 days (2d-iAVLs, 7d-iAVLs, 14d-iAVLs, and 28d-iAVLs; *n* = 3, for each implantation time). Briefly, the subdermal model was produced subjecting nAVLs to mild fixation with 0.625% (*w*/*v*) glutaraldehyde prior to implantation for progressive times as above into pouches formed by blunt dissection of the interscapular subdermis of 3-week-old male Sprague Dawley rats. No animals were killed specifically for the purpose of this study, since stocks of paraffin-embedded (or resin-embedded, see below) AVLs had been saved in the course of our previous investigations [24,25,26,27].

### 4.2. Immunohistochemical Assays

Histological sections of both nAVLs and 2d- to 28d-iAVLs were deparaffinised, re-hydrated, and incubated with (i) 0.1% Triton X-100 for 10 min, (ii) 3% hydrogen peroxide for 5 min, and (iii) 3% normal serum for 40 min. Additional incubation for 90 min at room temperature was performed with the following primary antibodies: 1:100 rabbit anti-cPLA2α (abcam, Cambridge, UK); 1:70 mouse anti-vimentin (Santa Cruz Biotechnology, Dallas, TX, USA); 1:15 mouse anti-ASMA (Chemicon International, Waltham, MA, USA); 1:50 mouse anti-CD68 (US Biological, Salem, MA, USA); 1:800 mouse anti-IL-6 (abcam, Cambridge, UK); 1:100 mouse anti-OPN (Santa Cruz Biotechnology, Dallas, TX, USA); 1:70 rabbit anti-fetuin-A (Novus Biologicals, Centennial, CO, USA); 1:25 goat anti-Anx-V (Santa Cruz Biotechnology, Dallas, TX, USA); 1:100 rabbit anti-cleaved caspase-3 (Cell Signaling, Danvers, MA, USA); 1:50 rabbit anti-MAP1 (Novus Biologicals, Centennial, CO, USA). Sections were then suitably incubated with 1:600 anti-rabbit (Jackson ImmunoResearch, Ely, UK), 1:100 anti-mouse (Santa Cruz Biotechnology, Dallas, TX, USA), or 1:400 anti-goat (Santa Cruz Biotechnology, Dallas, TX, USA) peroxidase-conjugated secondary antibody for 30 min. Peroxidase activity was detected using DAB chromogen (BioGenex, Fremont, CA, USA). As endogenous controls, primary antibodies were replaced with normal serum. After mild counterstaining with hematoxylin, histological sections were de-hydrated, soaked in xylene, and mounted with Eukitt® mounting medium. Image recording was carried out using an AxioImager photomicroscope (Carl Zeiss, Oberkochen, Germany).

### 4.3. Von Kossa Silver Staining for Calcium Binding Site Detection

Deparaffinised and re-hydrated histological sections of both nAVLs and 2d- to 28d-iAVLs were incubated with 1% silver nitrate for 15 min under direct sunlight. After rinsing with distilled water, sections were incubated with 5% sodium thiosulphate for 5 min, rinsed again, and mildly counterstained with hematoxylin. Sections were then de-hydrated, soaked in xylene, and mounted with Eukitt® mounting medium. Image recording was carried out using the Zeiss AxioImager photomicroscope as above.

### 4.4. Transmission Electron Microscopy

Additional samples excised from nAVLs and 2d- to 28d-iAVLs were fixed with a 25 mM sodium acetate/acetic acid buffer, pH 4.8, containing 2.5% glutaraldehyde, 0.05% phthalocyanine cuprolinic blue (Electron Microscopy Sciences, Hatfield, PA, USA), and 0.05 M magnesium chloride overnight at room temperature. Samples were then post-fixed with 2% osmium tetraoxide (Agar Scientific, Stansted, UK), dehydrated with graded ethanol solutions, and embedded in Epon-Araldite resin. Ultrathin sections were collected onto formvar-coated 2 × 1-mm-slot copper grids and contrasted with uranyl acetate and lead citrate. Observations and image recording were carried out using a CM12 STEM electron microscope (Philips, Eindhoven, The Netherlands).

### 4.5. Post-Embedding von Kossa Reaction

Semithin sections obtained from Epon-embedded samples as above were treated as previously described [24]. Sections were incubated with 1% silver nitrate for 15 min under direct sunlight and 5% sodium thiosulfate for 5 min, keeping glass slides on an 80 °C warm plate. Then, topless conic BEEM capsules were glued onto the glass slides so encircling each reacted semithin section and filled with Epon-Araldite fluid for section re-embedding. Once detached from the glass slide, re-embedded sections were cut to obtain ultrathin sections which were collected onto formvar-coated 2 × 1-mm-slot copper grids and weakly contrasted with uranyl acetate and lead citrate. Observations and image recording were carried out using the Philips CM12 STEM electron microscope as above.

### 4.6. Immunogold Labelling

Additional samples excised from nAVLs and 2d- to 28d-iAVLs were fixed with 4% paraformaldehyde, dehydrated with graded ethanol solutions, and embedded in LR-White resin. Ultrathin sections were incubated with (i) 5% normal serum for 30 min, (ii) 1:100 rabbit anti-cPLA2α (abcam, Cambridge, UK), 1:100 anti-mouse OPN (Santa Cruz Biotechnology, Dallas, TX, USA), or 1:25 anti-goat Anx-V (Santa Cruz Biotechnology, Dallas, TX, USA) primary antibody overnight at +4 °C, and (iii) 1:30 anti-rabbit (Jackson Immunoresearch, Ely, UK), 1:15 anti-mouse (Jackson Immunoresearch, Ely, UK), or 1:15 anti-goat (Jackson Immunoresearch, Ely, UK) gold-conjugated secondary antibody for 60 min at room temperature. As endogenous controls, primary antibodies were replaced with normal serum. After weak contrasting with uranyl acetate and lead citrate, observations and image recording were carried out using the Philips CM12 STEM electron microscope as above.

## Figures and Tables

**Table 1:** Positivity for calcification-related, inflammation, and cell death markers in native and experimentally calcified porcine aortic valve leaflets subjected to subdermal implantation in rats for 2 to 28 days.

	nAVL	2d-iAVL	7d-iAVL	14d-iAVL	28d-iAVL
**cPLA2α**	±	±	++	+++	++++
**IL-6**	−	−	−/±	±	+
**OPN**	±	±	++	+++	++++
**Fetuin-A**	±	−	−	−	−
**Anx-V**	±	±	+	+	+
**cl-C3**	−	−	−	−	−
**MAP1**	−	−	−	−	−

cPLA2α, calcium-dependent cytosolic phospholipase A2α; IL-6, interleukin-6; OPN, osteopontin; Anx-V, annexin-V; cl-C3, cleaved caspase-3; nAVL, native aortic valve leaflets; iAVL, aortic valve leaflets subdermally implanted for 2 to 28 days (d).

## Data Availability

Not applicable.
